# Modulation of the goodness of fit in hydrological modelling based on inner balance errors

**DOI:** 10.1371/journal.pone.0260117

**Published:** 2021-11-18

**Authors:** Francisco Pellicer-Martínez, Francisco Gomariz-Castillo, María Manuela Portela, Isabel María Martínez-Alcalá, José Miguel Martínez-Paz

**Affiliations:** 1 Department of Civil Engineering, Catholic University of Murcia (UCAM), Murcia, Spain; 2 Department of Geography, University of Murcia, Murcia, Spain; 3 Instituto Superior Técnico (IST), Lisbon University, Ceris, Lisbon, Portugal; 4 Department of Applied Economics, University of Murcia, Murcia, Spain; Soil and Water Resources Institute ELGO-DIMITRA, GREECE

## Abstract

In hydrological modelling, a good result for the criterion of goodness of fit does not always imply that the hypothesis of mass conservation is fulfilled, and models can lose their essential physical soundness. We propose a way for detecting this anomaly by accounting the resulting water balance during model simulation and use it to modulate the obtained goodness of fit. We call this anomaly in water balance as “inner balance error of the model”. To modulate the goodness of fit values, a penalty function that depends on this error is proposed. In addition, this penalty function is introduced into a multi-criteria objective function, which is also tested. This procedure was followed in modelling the Headwater of the Tagus River (Spain), applying the monthly abcd water balance model. Modulation of the goodness of fit allowed for detecting balance errors in the modelling, revealing that in the simulation of some catchments the model tends to accumulate water in, or release water from, the reservoir that simulates groundwater storage. Although the proposed multi-criteria objective function solves the inner balance error for most catchments, in some cases the error cannot be corrected, indicating that any error in the input and output data is probably related to groundwater flows.

## Introduction

For accurate hydrological planning, it is essential to know the spatial-temporal distribution of water resources in the basins in question [1]. This can be accomplished by naturalising flow records [2] and/or by using hydrological modelling [3]. At present, the use of hydrological models is the most-widespread practice, as they also can forecast future water resources under diverse land use and climate change scenarios [4]. They are therefore very useful tools for the authorities responsible for water resources management in order to adapt hydrological planning to predictable fluctuations in water availability [5].

These models recreate the water cycle by using mathematical formulations to simulate the hydrological processes that dominate the hydrology of a given catchment [6]. The water balance equation is applied to one or several reservoirs representing different natural water storage types (soil moisture, vegetation, aquifers), whose inputs and outputs are specified by mathematical expressions. This balance is computed at each of the time intervals (usually on a daily, weekly, or monthly scale) into which the input time series is divided—generally covering several years. The global water balance at the end of the simulated period should be close to zero [7].

Hydrological models are a tool that artfully combines available observations with our fundamental knowledge to describe the behaviour of the system through the implementation of scientific methods [8]. For this reason, several series of observations are necessary for them to be properly developed. Some variables (mainly climatic, edaphological and geometric) are used as input data and others (naturalized flows, piezometric and lake levels, accumulated snow heights, among others) as control data to determine the values of the parameters in the mathematical expressions that reproduce the main hydrological processes through the conceptual reservoirs of the models [9]. The values of these parameters are set to minimise the difference between the control data series simulated by the model and the real data.

This process of parameter determination is called model calibration [10–12]. While there are several procedures that can be used to carry out this process, it is generally done by minimizing an objective function that summarise the differences between the observed and the simulated values [13]. Many types of objective function can be found in the literature [7,14,15], but most are composed of “least squares” metrics [14], such as the Mean Square Error (MSE) [16] or the Nash-Sutcliffe Efficiency (NSE) [17]. However, some variants have been proposed to reduce the underestimation of temporal variability introduced by the above, such as Kling-Gupta Efficiency (KGE) [18,19] or the rational performance criterion (LME) [20]. Other common functions related to the water balance recreated by the model are bias (relative volume error) or Volumetric Efficiency (VE) [13]. The choice of function depends on the main aim of the study in which it is to be used [21,22]. Those functions based on “least squares” metrics force models to better reproduce the peak flows at the expense of low flows [17,23], while those based on the water balance force the model to reproduce the whole volume of the output series [24]. It is also possible to calibrate the model through a multi-criteria objective function that combine the aforementioned functions [7,21,25]. It is even possible to include streamflow characteristics in the objective function [26]. Besides, it must also be taken into account that some of the previous functions are mathematically related, e.g., NSE and MSE. However, there are more hidden relationships, as revealed by Gupta el al. [18] when they decompose the NSE and MSE functions. So, even when a single function is applied, several different targets are being addressed with pre-set weights. This explains why, in basins of high variability, high NSE values are usually associated with errors in the balance (bias). To solve this, Lindström [27] proposed a multi-criteria function in which a percentage of the bias committed is subtracted from the NSE value. In this respect, applying only one criterion is not sufficient for evaluating the consistency of a model, a topic currently considered to be of great importance [28]. This is because the hypothesis that no single objective function can represent all relevant characteristics of even one specific hydrological variable [25]. To address this problem, more alternatives have been proposed; for example, those based on a combination of more than one objective function (multi-objective calibration) to improve the robustness of the optimization. Thus, Huo and Liu [29] proposed a framework to compare the combination of several objective functions with the use of just one, demonstrating that a combination of two provides more complete and reliable dominant options than any single objective function. Other authors, such as Tian et al. [25], propose the optimization of a single objective function that can simultaneously address multi-response modes based on the composite likelihood index of four objective functions.

Most of the objective functions used in calibration are formulated in relative terms and also serve to evaluate the model goodness of fit (NSE, VE, KGE or bias). Therefore, they are also used to assess the quality of the simulation, and/or to select the model that best recreates the flow in a catchment. However, good simulation by the model does not always imply the correct modelling of the catchment hydrology. Such is the case when the global water balance resulting from the model is not close to zero [7], so the model lacks its essential soundness, and the predicted series are not reliable. Despite the existence of many objective functions, none of them ensures that the model meets this requirement that is assumed in every hydrological model, which is a situation to avoid. So, nothing prevents a high goodness of fit statistics from being accompanied by an erroneous water balance. One reason for this is the equifinality of the model parameters [30], meaning that different groups of parameters can often provide a similar goodness of fit. This can happen when the model structure is not suitable for the hydrological processes that actually occur in the simulated basin. Examples of this erroneous specification are: unregistered water extractions [31], groundwater exchanges between catchments [32], or erroneous data, such as the overestimation or underestimation of evapotranspiration [33].

The main objective of this work is to introduce the resulting water balance that a hydrological model makes during its simulation as a criterion for evaluating the goodness of fit and, also, as a criterion in the calibration process. For this purpose, the calculation of this water balance is defined and expressed in relative terms with respect to the observed output flow, in order to standardize this value. This water balance is called herein the inner balance error (ε) of the model and is expected to be close to zero. From this balance error, a penalty dimensionless function ϕ(ε) (between 0 and 1) which begins to reduce the value of goodness of fit obtained when this balance error is other than zero is proposed. The obtained goodness of fit is modulated according to this balance error. In addition, this penalty function (ϕ(ε)) is tested in the calibration process. In this sense, a multi-criteria objective function which forces to the model to reduce its inner balance error is proposed. Thus, the group of sets of parameters that are obtained with this multi-criteria objective function is reduced to those that, in addition to providing a high goodness of fit, commit a low inner balance error. In cases where inner balance error remain high, the water balance error is not solved, indicating that the model is incorrectly specified, either because of its structure or because of incorrect input and output data. The use of this penalty function to model the Headwater of the Tagus River Basin (Spain) using a monthly lumped water balance semi-distributed model. This basin is one of the most important in the Iberian Peninsula in terms of water resources management, since, in addition of being the head of one of its most-important rivers, it is the origin of the largest inter-river water transfer in Spain: the Tagus-Segura transfer.

## Materials and methods

The methodology is ordered sequentially to facilitate the connection between the different sub-sections. First, the inner balance error and the penalty function are defined; second, the objective functions used in this work are presented; third, the study area is described; fourth, the data and specifications to be used in the calibration-validation are detailed; and, finally, the model used and the criteria used for its choice are indicated.

### Inner balance error and penalty function

The inner balance error is based on the water balance (Δ*V*) between input and output data of the catchment during the simulation of the model. Precipitation (P_i_) is the input variable, while the actual evapotranspiration (ET_i_) and the simulated flow (Q_s,i_) are the output variables. If a catchment receives flow from another upstream catchment, then this flow is added as input in the balance (Q_u,i_).This balance is defined in Eq 1 for a period of *n* time intervals. A positive value of inner balance indicates that the model stores water, while a negative value indicates that the model releases more water than enters in it.


$$\Delta V=\sum_{i=1}^{n}P_{i}-\sum_{i=1}^{n}{ET}_{i}-\sum_{i=1}^{n}Q_{s,i}+\sum_{i=1}^{n}Q_{u,i}$$ (1)

The resulting water balance is divided by the observed flow (Q_o,i_) in the analysed catchment so that it can be expressed as a percentage (Eq 2). In this way, the inner balance error (ε) is a statistic that can be interpreted. If ε > 0, the catchment stores water, and its value indicates the amount of stored water related to the observed flow. For example, if ε = 0.25, this indicates that the catchment stores, on average for each time interval, an amount of water equal to a quarter of the flow that it releases. So, there is an imbalance in the inner water balance equivalent to 25% of the observed flow. If ε < 0, then the flow released from the catchment is higher than the input water. The latter situation is only possible if the model parameters that represent the initial stored water have no limit. Although this is not desirable, not restricting this parameter can sometimes provide better hydrological simulations that give a clue to real hydrological aspects of the basin under study.


$$\varepsilon =100\cdot \left[\frac{\sum_{i=1}^{n}P_{i}-\sum_{i=1}^{n}{ET}_{i}-\sum_{i=1}^{n}Q_{i}+\sum_{i=1}^{n}Q_{u,i}}{\sum_{i=1}^{n}Q_{o,i}}\right]$$ (2)

This statistic is similar to the well-known Pbias, which measures the average tendency of the simulated data to be larger or smaller than their reference values. But, in this case, Pbias represents an external balance between observed (Q_o,i_) and simulated flow (Q_s,i_), without considering the internal behaviour of the model (Eq 3).


$$Pbias=100\cdot \left[\frac{\sum_{i=1}^{n}\left(Q_{o,i}-Q_{s,i}\right)}{\sum_{i=1}^{n}Q_{o,i}}\right]$$ (3)

The Pbias and the inner water error are similar statistics. The numerator in both equations is a water balance (Eqs 2 and 3), and they have the same denominator, representing the observed flow. For a proper simulation, both need to be close to zero and, their results are interpreted similarly. For example, a Pbias of 0.25 indicates that the model only reproduces 75% of the observed flow (and underestimates 25%). Hence, it indicates that there is an imbalance in the external water balance that is equivalent to 25% of the observed flow. Due to this similarity, the inner balance error is also denominated in this work as inner-Pbias. However, there is a small difference between these two statistics. While a Pbias equal to zero is always desirable, the balance error must, at least, be close to zero (it does not have to be equal to zero) since a degree of variation between wet and dry periods is always possible.

**Fig 1:**
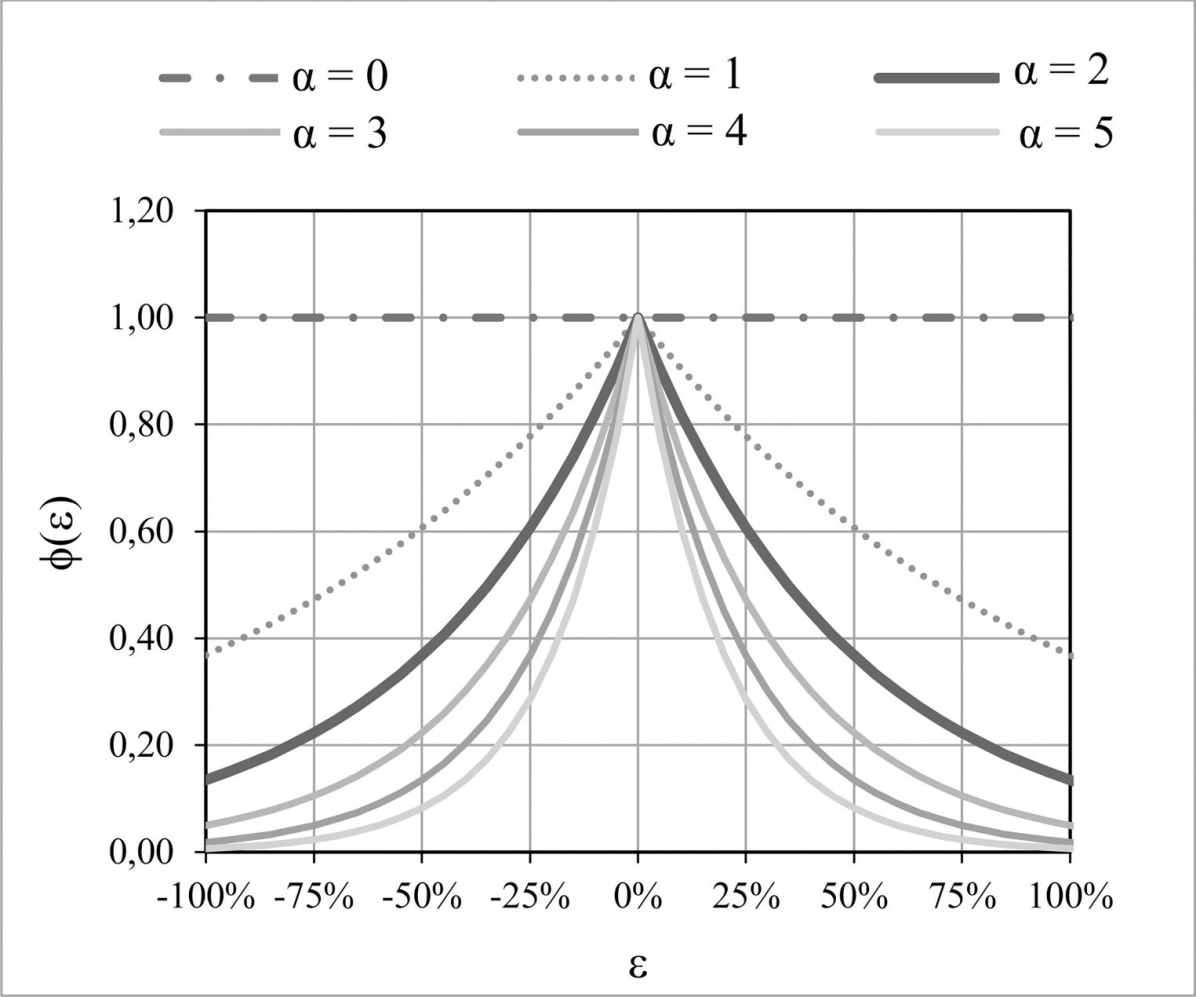
An example of the penalty function ϕ(ε) according to six values of the α-parameter.

The use of the most widely used statistics for calibration purposes and to evaluate the goodness of fit does not guarantee that the inner balance error is close to zero, since they usually focus on outlet flow. As a consequence, it is possible to simultaneously obtain a high goodness of fit and a severe inner water balance error, so we propose to use the inner balance error (ε) to modulate the goodness of fit. The quality of a modelling is generally measured by means of standardized statistics between 0 and 1 that seek a maximum: for example, NSE, VE or KGE. Hence, an exponential penalty function ϕ(ε) that depends on ε and on one parameter (α) is proposed (Eq 4). This function also standardizes the error for values between 0 and 1 (Fig 1), so, if a simulation complies with the mass conservation equation (ε = 0) there is no penalisation to the statistic used to indicate the goodness of fit (ϕ(ε) = 1). Otherwise, if the simulation commits an inner balance error (ε≠ 0), then the goodness of fit is reduced (ϕ(ε) < 1).


$$\phi (\varepsilon )={exp}^{-\alpha \cdot |\varepsilon |}$$ (4)

The proposed function ϕ(ε) is the solution of a linear differential equation in which the variation of the penalty according to the inner balance error committed is proportional to the latter (ε). This implicitly means that the relative variation in the penalty is proportional to the variation in the inner balance error (see Eq 5). The advantages of ϕ(ε) include that it is a easily adjustable continuous function. Bearing this in mind, the value of the α-parameter can be defined in such a way as to pre-establish a limit for the inner balance error. If the objective is to ensure the detection of errors of 100% magnitude, which implies that this error is equal to the observed outlet flows, the α-parameter must be around 4 (Fig 1); thus, errors close to or higher than 100% will result in a value of zero. If the α-parameter selected is equal to zero, there is no penalty, since ϕ(ε) is always equal to 1.


$$\frac{d\phi (\varepsilon )}{d\varepsilon }=\alpha \cdot \phi (\varepsilon )\to \frac{d\phi (\varepsilon )}{\phi (\varepsilon )}\propto \alpha \cdot d\varepsilon \to \frac{\Delta \phi }{\phi }\propto \alpha \cdot \Delta \varepsilon$$ (5)

### The objective functions used

The objective function chosen to calibrate the model is the NSE coefficient (Eq 6), which is one of the most widely used statistics to measure the goodness of fit of hydrological models [17,34]. The NSE coefficient takes values between (-∞ - 1]; the closer to 1, the better the model’s performance [16].


$$NSE=1-\frac{\sum_{i=1}^{n}{\left(q_{o,i}-q_{s,i}\right)}^{2}}{\sum_{i=1}^{n}{\left(q_{o,i}-\bar{q_{o}}\right)}^{2}}$$ (6)

In this equation, q_s,i_ and q_o,i_ are, respectively, the *n* simulated flows and the *n* previously naturalized observed flows [2] that were transformed by the square root function (Eq 7). Thus, the errors obtained in the modelling (u_t_) are stochastically independent and distributed normally with a mean of zero (Eq 7) [35]. The value of $\bar{q_{o}}$ is the mean of the series q_o_,_i_.


$$\begin{array}{c} q_{s,i}=\sqrt{Q_{s,i}}\\ q_{o,i}=\sqrt{Q_{o,i}} \end{array}\}\;q_{o,i}=q_{s,i}+u_{t};\;u_{t}=N[0;\sigma^{2}];\;E[u_{t};u_{t-1}]=0$$ (7)

In addition to NSE, which is calculated below by undoing the square root transformation, other goodness of fit statistics were calculated (also by undoing the square root transformation) in order to support the results provided by NSE. This provides a set of metrics that leads to a broader assessment of the capacity of the simulation [22], as different metrics can be sensitive in different ways to the same errors [15]. The goodness of fit statistics we used were VE [13], KGE [18], and Pbias [13]. The modulation by ϕ(ε) was applied to NSE, VE and KGE, in order to identify the catchments with high inner water balance errors. Here, we only show how the penalty function is applied to NSE, since it would be similar for the other two functions (Eq 8).


$${NSE}^{*}=\phi (\varepsilon )\cdot NSE$$ (8)

Finally, to ascertain whether it is possible to simultaneously obtain a high goodness of fit and an inner balance error close to zero, Eq 8 is used as objective function in the calibration. This multi-criteria objective function has two goals, to maximise the criterion of goodness of fit, and to minimise the inner balance error (ε). Both goals are weighted, at first glance, by the α-parameter. But, when this new multi-criteria objective function is analysed, as ε values close to zero are expected, the first degree Taylor polynomial of ϕ(ε) is obtained, that is, ϕ(*ε*)≅1−*α*|*ε*|. Now, when applying this polynomial to NSE, the resulting function is similar to that proposed by Lindström [27], where NSE is penalized by the Pbias multiplied by the weight “*w*” (Eq 9). But, in the function used here, the weight “*w*” of the penalty given by inner-Pbias (ε) is the α-parameter and also the NSE itself, which depends on the simulation (Eq 10). This implies that the penalization will be greater for models with both large ε (inner-Pbias) and high NSE values that are not congruent with the water balance done by the hydrological model. If *α* = 1, then *w* = NSE (Eq 11).


$${NSE}^{\prime }=NSE-w|Pbias|$$ (9)

$${NSE}^{*}≅NSE-\alpha \cdot NSE|\varepsilon |$$ (10)

$${NSE}^{*}≅NSE-NSE|\varepsilon |$$ (11)

### The Headwater of the Tagus River Basin

**Fig 2:**
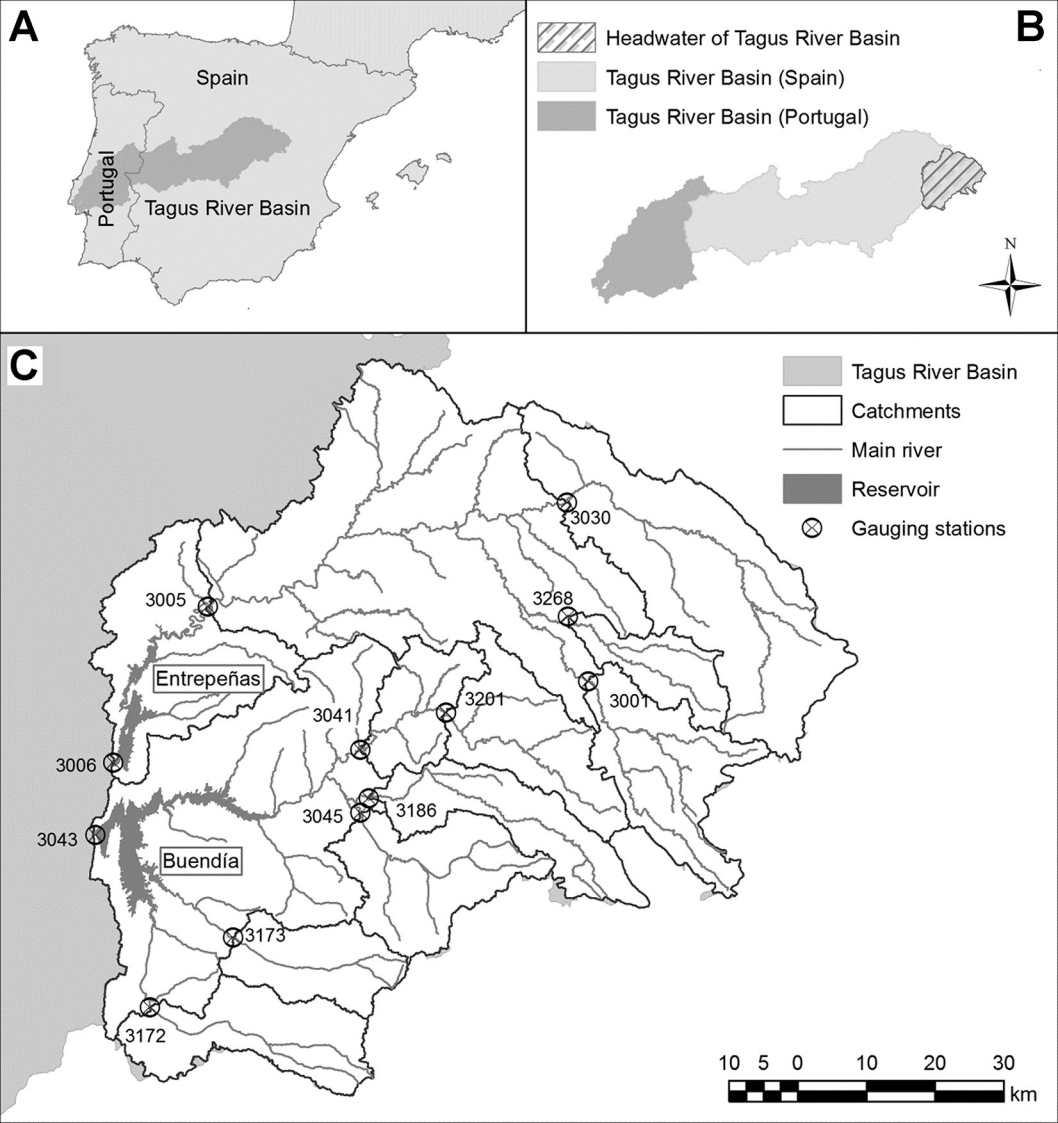
Study area. Location of the Tagus River Basin (A). Location of the Headwater of the Tagus River in Spain (B). Main streams in the Headwater of the Tagus River, location of the gauging stations, geographical delimitation of the catchment with flow observations and location of the Entrepeñas and Buendía reservoirs (C). Source: BTN25 2006–2019 CC-BY 4.0 ign.es (for administrartive data); CC-BY 4.0 © Ministerio para la Transición Ecológica y el Reto Demográfico (for river basins, main rivers, reservoirs and gauging stations); Derived data from MDT25 2015 CC-BY 4.0 ign.es (for Catchments).

The Headwater of the Tagus River Basin is located in the centre of the Iberian Peninsula (Fig 2A and 2B). It has an area of about 10,000 km^2^ and a high-mountain Mediterranean climate with a marked seasonality between the summer and winter periods [36]. The average annual rainfall is 620 mm, being minimal in the summer months. The average temperature is 11°C, with minima below zero in the winter, and maxima in summer higher than 30°C [4]. Finally, the hydrogeology is dominated by carbonate aquifers shared with the other river basins, such as the Guadiana and the Júcar Rivers [37].

The water uses are urban, industrial and irrigation, which together represent a very small percentage of the available water resources since the area has a low population density and is not conducive to agriculture. There are numerous hydropower stations and an important thermonuclear power station, which do not consume water. However, the water resources generated within the basin are essential for other uses located downstream that require high volumes of water: electricity production, agriculture, urban supply (including the city of Madrid) and the maintenance of environmental flows. In addition, a substantial part of these water resources is diverted to the Segura River Basin by transfer of the water stored in the main two reservoirs (Entrepeñas and Buendía) located within the study area (Fig 2C).

### Datasets and specifications

The data used in the models (abcd and the other two presented in the next point) came from several official Spanish sources. 1) the Digital Terrain Model with a resolution of 25 m, was produced by the National Geographic Information Centre [38]; 2) the river gauging stations and the river flow observations used to calculate the natural flows are part of the database of the Integrated Network of Gauging Stations of the Hydrographical Studies Centre of Spain [39,40]; 3) the series of precipitation and potential evapotranspiration were obtained from monthly raster maps created by the Ministry of Agriculture, Food and Environment [41,42].

To apply the models, the study area was divided into the 12 catchments defined by the river gauging stations (Fig 2C), based on which a semi-distributed model was constructed. The period covered by observations in all the gauging stations ran from October 1982 to September2010, with 336 monthly values available for each variable. The series of precipitation and potential evapotranspiration values in each catchment were obtained by averaging the values of the cells located within it. For each catchment (identified by its code, also included in Fig 2C), Table 1 lists its area and the average yearly values of the main meteorological variables, as well as of the natural flow.

**Table 1:** Main physical and hydrological features of the catchments in the Headwater of the Tagus River Basin.

Name	Peralejos	Ventosa	Taravillas	Trillo	Entrepeñas	Priego Escabas	Huete	La Peraleja	Priego Trabaque	Molino de Chincha	Alcantud	Buendía
**Code**	3001	3030	3268	3005	3006	3045	3172	3173	3186	3201	3041	3043
**River**	Tagus	Gallo	Cabrillas	Tagus	Tagus	Escabas	Mayor	Guadamejud	Trabaque	Guadiela	Guadiela	Guadiela
**Area (km** ^**2**^ **)**	412	943	183	1720	570	327	360	257	388	363	211	1416
**Precipitation (mm/year)**	792	552	696	624	576	768	540	516	636	816	708	528
**ETP (mm/year)**	588	660	624	648	708	648	780	780	720	624	648	756
**Temperature (°C)**	10	10	9	11	12	11	13	13	12	10	11	13
**Q (mm/year)**	325	53	170	134	49	330	38	28	38	234	397	58

Cascade calibration [43], which consists of determining the parameters for different catchments, simulating from upstream to downstream, was used to determine the model parameters. The values of the parameters calibrated for the catchments located upstream in each river section are considered fixed when calibrating the catchments downstream. The first available natural observed flow values are used as “take-off” in each model, while the remaining *n* observations are divided into two periods: the first, with two-thirds of the remaining *n* observations, is used for calibration, and the second for validation [44]. This cascade calibration was carried out with the GRG2 nonlinear optimisation algorithm [45], which looks for the extreme values of the functions using the generalised reduced gradient algorithm [46].

### Water balance model selected: Abcd model

A previous study was made, using three of the most widely used lumped water balance models: the abcd [47], the Thornthwaite-Mather [48], and the GR2 [49] models. All of them simplify the water cycle into two reservoirs: one that simulates the soil moisture balance (S), and another that represents the aquifers (G) where groundwater discharges occur. The input for all three models is precipitation (P) and the outputs are actual evapotranspiration (ET) and flow (Q). Detailed information about the structure and equations of these models is presented in S1 Appendix. The NSE statistic was used as criterion to select the model. The abcd model provided the highest and most consistent values of NSE and was consequently selected to analyse the use of inner water balance (S2 Appendix.).

## Results

This section is structured as follows. First, the results of the simulation using NSE as objective function are presented, and the penalty function is applied to NSE, VE and KGE. Next, the previously defined multi-criteria objective function is used (NSE*). For this, an analysis is performed to define the most appropriate value of the α-parameter to include the reduction of the inner balance error as a calibration goal.

### Modulation of the goodness of fit using NSE as objective function

The model was calibrated to maximise the NSE calculated with square root transformed data. Table 2 shows the main results of the modelling, both for the calibration and validation periods. The top part of each period includes the main statistic generally used to assess the goodness of fit of a hydrological model (NSE, VE, KGE, RMSE and Pbias). The results related to water balance are presented below: the inner balance error (inner-Pbias), the resulting water balance in model reservoirs (ΔS: soil moisture, and ΔG: groundwater), and the resulting water balance model both in absolute terms (ΔV) and in relative terms with respect to the average observed flow (as the inner balance error). These water balances were calculated as the difference between the water stored in the reservoirs at the beginning and at the end of the simulation (just after the “take-off” period). The final part contains the results of the NSE, VE and KGE indicators modulated by the penalty function for a value of α = 4. This value of α is subsequently analysed.

**Table 2:** Main indicators for the calibration of the abcd model, using NSE as objective function.

	Indicators	Catchment Code											
		3001	3030	3268	3005	3006	3045	3172	3173	3186	3201	3041	3043
**Calibration**	**NSE**	0.77	0.39	0.69	0.71	0.75	0.82	0.73	0.76	0.57	0.84	0.74	0.86
	**VE**	0.68	0.70	0.72	0.74	0.77	0.80	0.73	0.60	0.51	0.76	0.72	0.81
	**KGE**	0.76	0.45	0.65	0.73	0.74	0.78	0.85	0.71	0.33	0.78	0.70	0.85
	**RMSE (Mm** ^**3**^ **/month)**	5.3	2.0	1.1	15.3	15.8	3.1	0.7	0.4	1.2	4.2	8.2	10.2
	**Pbias (%)**	-4%	8%	6%	7%	3%	2%	3%	2%	11%	2%	3%	0%
	**ε: Inner–Pbias (%)**	3%	400%	79%	43%	0%	-4%	189%	262%	355%	92%	-32%	-2%
	**ΔS (Mm** ^**3**^ **)**	49	62	16	123	57	25	22	14	24	31	20	90
	**ΔG (Mm** ^**3**^ **)**	-52	3423	437	3628	-8	-24	480	303	967	1353	-494	-86
	**ΔV (Mm** ^**3**^ **)**	-3	3486	453	3751	49	1	502	318	992	1384	-475	4
	**Balance Error (%)**	0%	418%	85%	49%	1%	0%	212%	267%	368%	117%	-29%	0%
	NSE*	0.68	0.00	0.03	0.13	0.74	0.71	0.00	0.00	0.00	0.02	0.20	0.81
	VE*	0.60	0.00	0.03	0.13	0.75	0.70	0.00	0.00	0.00	0.02	0.20	0.76
	KGE*	0.67	0.00	0.03	0.13	0.72	0.67	0.00	0.00	0.00	0.02	0.19	0.80
**Validation**	**NSE**	0.59	0.34	0.49	0.59	0.68	0.76	0.63	0.49	0.70	0.77	0.75	0.70
	**VE**	0.64	0.63	0.63	0.68	0.72	0.78	0.56	0.38	0.60	0.71	0.71	0.75
	**KGE**	0.77	0.45	0.54	0.69	0.79	0.86	0.63	0.27	0.79	0.86	0.62	0.80
	**RMSE (Mm** ^**3**^ **/month)**	5.9	2.1	1.4	17.5	16.0	2.6	0.7	0.7	0.6	2.6	5.9	11.3
	**Pbias (%)**	-14%	-6%	0%	-6%	-12%	-10%	-19%	-6%	-20%	-6%	2%	-13%
	**ε: Inner–Pbias (%)**	14%	394%	60%	50%	1%	13%	239%	260%	458%	102%	-16%	17%
	**ΔS (Mm** ^**3**^ **)**	-20.1	-17.5	-5.7	-20	-2.2	-4.4	4.2	6.1	3	-6.7	-6.3	13.0
	**ΔG (Mm** ^**3**^ **)**	21.8	1560	154	1533	37.1	27.7	257	154	449	615.2	-170.8	107.1
	**ΔV (Mm** ^**3**^ **)**	1.7	1543	148	1513	34.9	23.3	261	160	452	608.5	-177.1	120.1
	**Balance Error (%)**	0%	388%	61%	44%	1%	3%	220%	254%	437%	96%	-14%	4%
	NSE*	0.59	0.00	0.04	0.10	0.65	0.45	0.00	0.00	0.00	0.01	0.40	0.35
	VE*	0.64	0.00	0.06	0.12	0.70	0.47	0.00	0.00	0.00	0.01	0.38	0.38
	KGE*	0.77	0.00	0.05	0.12	0.76	0.52	0.00	0.00	0.00	0.01	0.33	0.40

**Fig 3:**
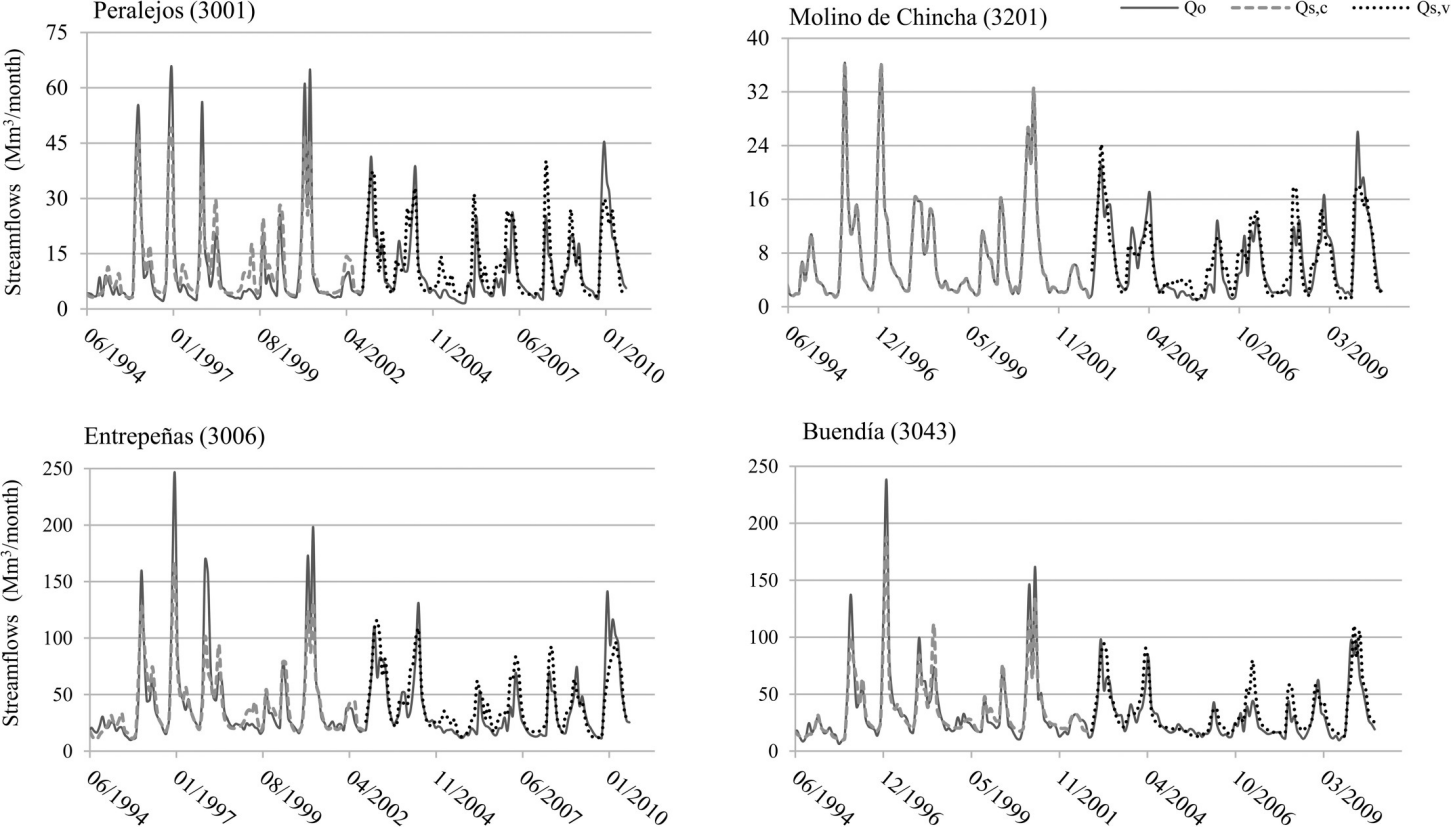
Observed and simulated flow in four catchments. Qo stands for observed flow and Qs,c and Qs,v for simulated flow in the calibration and validation periods, respectively.

The values of NSE in most of the catchments were above 0.60 for both the calibration and validation periods. Only in one catchment (3030-Ventosa) was a value lower than 0.50 found. These results are similar to or even outperform those obtained with a distributed model in the same geographical area [4], or those produced by lumped water balance models in other geographical areas [50–52]. Of note is the fact that the simulation of the flows into the Entrepeñas and Buendía reservoirs, which are the main water resources management infrastructures within the study area, resulted in high NSE values (Table 2). Therefore, using this statistic, it can be affirmed that the model properly represents the flow in the main points of the study area [34,51], and could be used to estimate their water resources. As an example, Fig 3 shows a comparison between simulated and observed flow in four catchments (3001-Peralejos, 3201-Molino de Chincha, 3043-Buendía and 3006-Entrepeñas).

The resulting VE values were slightly lower than NSE because the latter was the objective function used and because VE is not based on "least squares". However, these VE values were also high and even more consistent in validation than NSE for all the catchments. So, the hydrological performance can be described as adequate. The obtained values for KGE were similar, although slightly lower, than NSE. In the Ventosa (3030) and Priego-Trabaque (3186) catchments the values were as low as those of NSE. But, in general, the results were mostly acceptable, and the values were higher in the main control points (the Entrepeñas and Buendía dams). The results of the RMSE and Pbias were acceptable in most of the catchments, and so do not conflict with the above results. It should be noted that all these results were, in general, similar for calibration and validation, which lends robustness to the modelling carried out. It should also be noted that for the same catchment, not all the used indicators provided the same quality of fit. In other words, there were catchments with high values of NSE and KGE, but which also had high RMSE values compared with those obtained for other catchments. This is particularly the case for catchments that receive water from other upstream catchments (3005, 3006, 3041 and 3043), since the calibration is not able to reduce the noise introduced by this input variable. This is one of the mistakes associated with cascade calibration. These results confirm the recommendations that several goodness of fit statistic should be used to assess a model’s performance. As pointed out by Krause et al. [23], the objective should be to provide good values for a set of measures to include all the dynamics of the results of the models.

**Fig 4:**
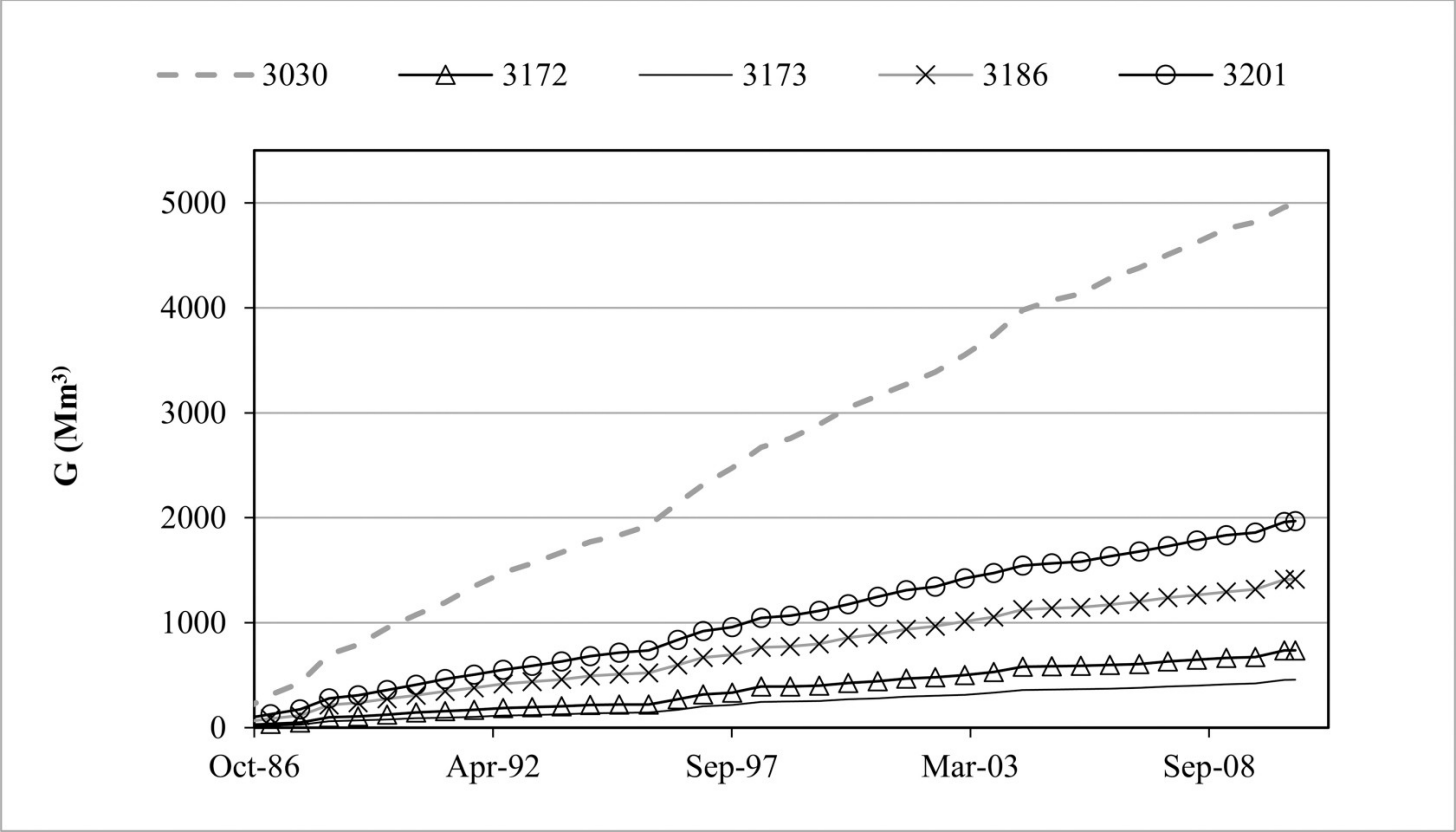
Evolution of stored water in the reservoir (G) simulating the aquifers in the catchments of Ventosa (3030), Huete (3172), La Peraleja (3173), PriegoTrabaque (3186), and Molino de Chincha (3201).

When the results of NSE, VE and KGE modulated with the penalty function are analysed using α = 4 (NSE*, VE*, and KGE*), it is clear that only in four catchments the model performs well using these criteria (3001-Peralejos, 3006-Entrepeñas, 3045-Priego Escabas and 3043-Buendía). The penalty was very severe in the rest of the catchments, providing values equal to zero in 4 cases (3030-Ventosa, 3172-Huete, 3173-La Peraleja and 3186-Priego Trabaque). This reduction in goodness of fit indicates that there is a balance error of around 100% or more in the modelling, given the value of the α-parameter used. In fact, these errors were between 189% and 400% (calibration period), which indicates that, for example, the water accumulated in the 3030 catchment is four times the observed flow. These inner balance errors persisted throughout the simulation in all catchments, since they were of the same order of magnitude in both the calibration and validation periods. The origin of these errors was mainly the stored water in the reservoir that simulates groundwater (G). The total balance error (ΔV) was practically the same as the groundwater balance error (ΔG), the same occurring for the total Balance Error (%) of the model when compared with the Inner-Pbias (ε). Thus, these high values of ε indicate that the parameters used for some catchments produced an inadequate representation of the hydrological processes that actually occur in them. This can also be seen when representing the stored water in aquifers (G) throughout the calibration and validation periods in some catchments with high ε, as exemplified in Fig 4. Among them, the most striking example was Molino de Chincha (3201), which provided one of the highest goodness of fit values (see Table 2), but whose stored water in G at the end of the calibration-validation was equal to the flow exiting the catchment during the whole simulation.

**Fig 5:**
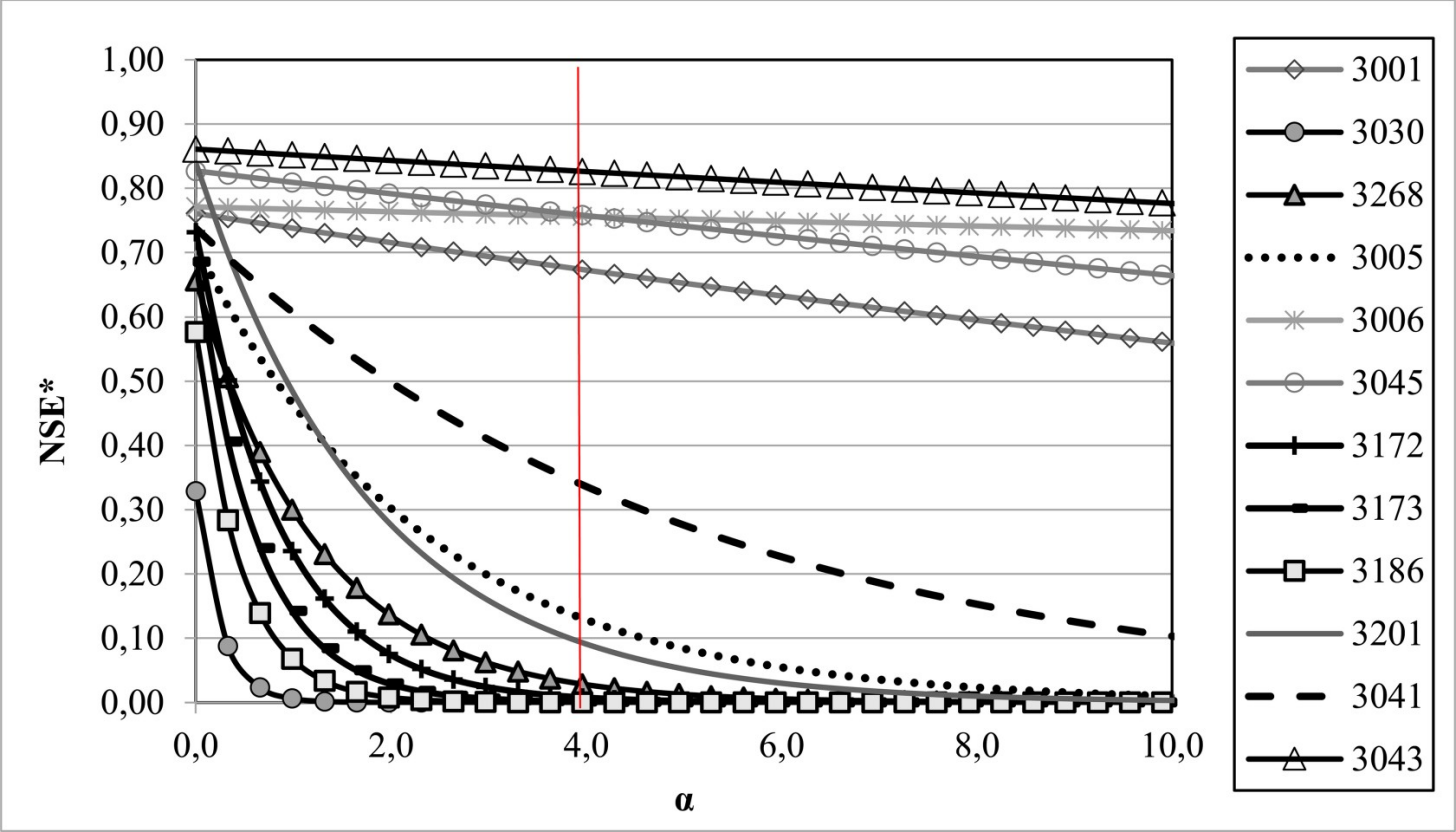
Analysis of the influence of the α-parameter on the modulation of the penalty function.

Fig 5 serves to analyse the optimum value of the α-parameter in the penalty function, showing how NSE is modulated according to the α-parameter used (NSE→NSE*). The intersections between the different curves with the red vertical line are the NSE* values shown in Table 2. The modulation is similar for the VE and KGE indicators, so it is not necessary to include a summary of them in a graph. In catchments whose simulations generate low inner balance errors, the decrease is almost linear with respect to the α-parameter and there are hardly any penalties. However, simulations with high inner balance errors, greater than 200%, an α-parameter equal to 2 is sufficient to produce an NSE value of 0. To automatically detect catchments with inner balance errors of around 100% (NSE* = 0), α-parameter = 4 is recommended, and for inner balance errors around 50%, α-parameter = 8 should be used, while an α-parameter equal to 10 would be even more restrictive (inner balance errors of around 25%). In this case, where only modulation of the goodness of fit is intended, the value of the α-parameter does not influence the calibration carried out, since it is applied later. Thus, the value can be easily modified, and it is possible to use a value as restrictive as deemed. For this specific case, α = 4 has been considered in order to detect the cases whose modelling significantly violates the principle in which the balance at the end of the model must be close to zero (as mentioned above, with inner balance errors of around 100%).

### Incorporating the penalty function ϕ(ε) into the objective function NSE

**Fig 6:**
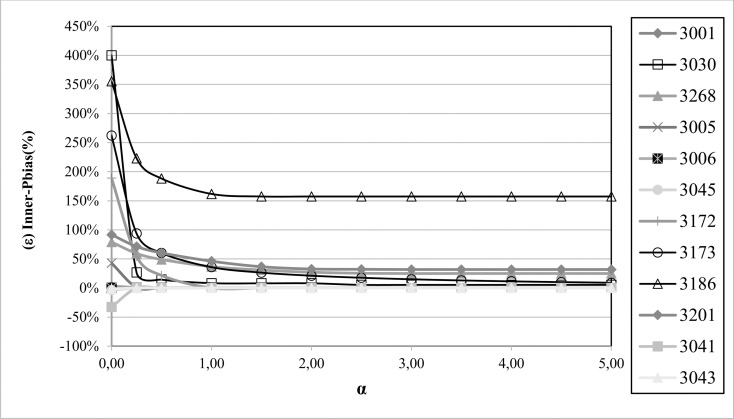
Inner balance error reduction according to the α-parameter used in the penalty function.

**Fig 7:**
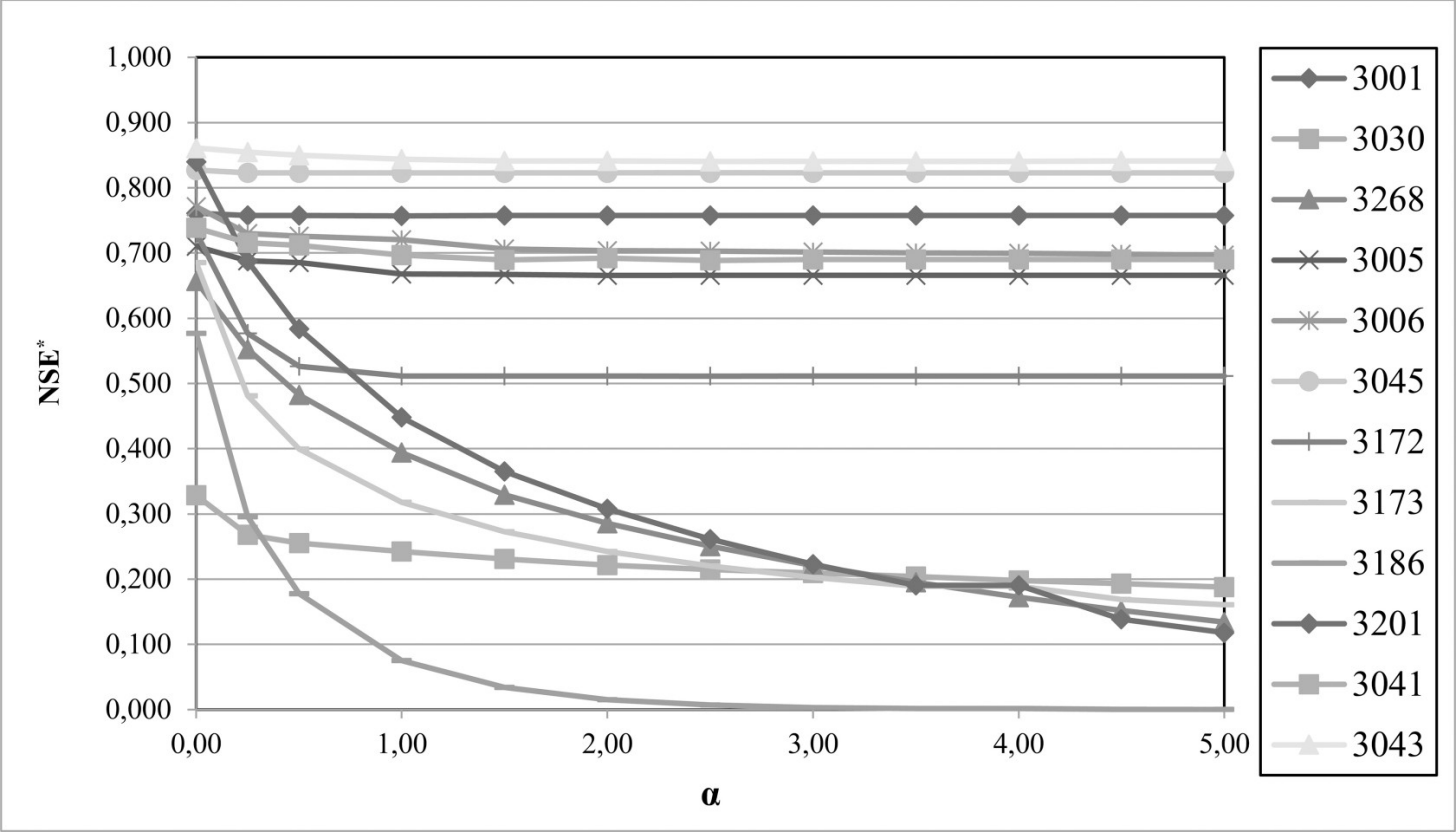
NSE* variation according to the α-parameter used.

The aim of introducing the penalty function as a part of the objective function is to force the model to reduce its inner balance error. But one question needs to be resolved first—the most appropriate value of the α-parameter to use in calibration. To answer this question, the hydrological model was recalibrated for the entire basin (12 sub-catchments), varying the α-parameter in the range [0–5]. When α = 0, the results are the same from the previous section, since there are no penalties (ϕ(ε) = 1), but they serve as starting point. In order to discern the most appropriate value, the inner balance error that occurs for each calibration was taken as a reference (Fig 6). Likewise, the value of the optimization function used (NSE*) was also calculated in order to verify that obtained values of the different optimizations were decreasing (Fig 7).

Fig 6 shows that the inner balance error was significantly reduced in all the catchments when using α = 1. For example, in catchment 3030 the error fell from 400% to 8%, and in another seven catchments the inner balancer error was reduced to 0%. Increasing α from 1 to 2 reduced inner balance errors slightly and stabilized them. Therefore, it would be sufficient to use α-parameter values of between 1 and 2 to significantly reduce the inner balance error of the modelling carried out or, at least, for the inner balance error (ε) to reach a value close to its minimum. In view of these results, it seems advisable to use α = 1 when the penalty function is included in the optimization (a value much lower than recommended to detect balance errors, which would be in the range 4–10). In this way, the penalty function can be reduced to Eq 12, and the objective function used would be related to Eq 11.


$$\phi (\varepsilon )={exp}^{-|\varepsilon |}$$ (12)

A higher value should be used for the α-parameter if the aim is to drastically reduce the inner balance error, but calibration efficiency would also be reduced. The maximum recommended value for the α-parameter is 2. As can be seen from Fig 6, using a higher value does not ensure that the balance error is reduced. In this sense, using a very high value for the α-parameter for catchments whose modelling has a natural or systematic inner balance error means that the objective function will always be around 0 and that it will be difficult to find a maximum for it.

Fig 7 shows that, in the catchments where the model achieves a null inner balance error, NSE* values do reach the stability. But, if the model is not capable to solve the inner balance error, the NSE* value decreases as the value of the α-parameter increases. So, increasing the α-parameter does not guarantee a readjustment of the parameter set that provides a null inner balance error.

Table 3 shows the results of the optimization using NSE* with the α-parameter equal to 1. Comparison of these with those obtained previously (Table 2) shows that there were hardly any differences with respect to the VE, RMSE and ΔS indicators, both in the calibration and validation periods. The Pbias values were similar in the calibration period, but in some catchments, it increased slightly and in others decreased. However, the only observed drawback was that the Pbias values increased slightly in the catchments in which it was not possible to reduce the balance error, especially for the validation period. The NSE values were practically the same, except in the catchments that showed the highest inner balance errors, which emphasizes the need of including the inner balance as a criterion in optimization. The KGE values were significantly reduced in Ventosa (3030) and Prieo-Trabaque (3186) catchments, while in others these statistics increased slightly, since it is not the optimization function used.

**Table 3:** Main indicators for calibration of the abcd model, using NSE* as objective function (*α* = 1).

	Indicators	Catchment Code											
		3001	3030	3268	3005	3006	3045	3172	3173	3186	3201	3041	3043
**Calibration**	**NSE**	0.76	0.28	0.62	0.67	0.70	0.82	0.35	0.49	0.42	0.73	0.71	0.85
	**VE**	0.68	0.71	0.69	0.72	0.75	0.80	0.65	0.46	0.43	0.67	0.70	0.80
	**KGE**	0.74	0.24	0.69	0.78	0.79	0.80	0.57	0.67	0.38	0.82	0.68	0.87
	**RMSE (Mm** ^**3**^ **/month)**	5.4	2.1	1.3	15.8	16.7	2.9	1.1	0.7	1.3	4.3	8.1	10.8
	**Pbias (%)**	-2%	5%	7%	4%	0%	-1%	2%	6%	15%	3%	3%	-1%
	**ε: Inner–Pbias (%)**	0%	6%	37%	0%	0%	0%	0%	36%	162%	46%	0%	0%
	**ΔS (Mm** ^**3**^ **)**	50	77	18	157	57	24	20	13	27	39	15	90
	**ΔG (Mm** ^**3**^ **)**	-74	102	220	184	-8	-19	30	42	454	658	70	-143
	**ΔV (Mm** ^**3**^ **)**	-24	179	238	340	49	5	51	54	481	697	85	-54
	**Balance Error (%)**	-1%	21%	45%	4%	1%	0%	21%	46%	178%	59%	5%	-3%
	NSE*	0.76	0.26	0.43	0.67	0.70	0.82	0.35	0.34	0.08	0.46	0.71	0.85
	VE*	0.68	0.66	0.48	0.72	0.75	0.80	0.65	0.32	0.09	0.42	0.70	0.80
	KGE*	0.74	0.23	0.48	0.78	0.79	0.80	0.57	0.47	0.08	0.52	0.68	0.87
**Validation**	**NSE**	0.58	0.01	0.39	0.48	0.57	0.71	0.45	0.50	0.15	0.59	0.71	0.59
	**VE**	0.64	0.55	0.58	0.63	0.67	0.75	0.43	0.28	0.23	0.59	0.65	0.69
	**KGE**	0.77	0.09	0.50	0.64	0.74	0.82	0.67	0.48	0.56	0.74	0.59	0.74
	**RMSE (Mm** ^**3**^ **/month)**	6.0	2.6	1.6	19.8	18.5	2.9	0.9	0.7	1.0	3.4	6.4	13.1
	**Pbias (%)**	-11%	-11%	-6%	-8%	-14%	-14%	-32%	-21%	-61%	-27%	-15%	-24%
	**ε: Inner–Pbias (%)**	11%	13%	18%	7%	1%	17%	59%	50%	235%	50%	16%	28%
	**ΔS (Mm** ^**3**^ **)**	-21.0	-24.9	-7.0	-43	-2.2	-4.2	6.4	8.8	0	-12.4	-3.7	13.2
	**ΔG (Mm** ^**3**^ **)**	25.6	33	36	9	37.1	25.2	26	10	180	160.1	17.6	104
	**ΔV (Mm** ^**3**^ **)**	4.6	8	29	-35	34.9	21.0	32	18	180	147.7	13.9	117
	**Balance Error (%)**	0%	2%	12%	-1%	1%	3%	27%	29%	174%	23%	1%	4%
	NSE*	0.58	0.01	0.35	0.48	0.57	0.60	0.25	0.30	0.01	0.36	0.60	0.45
	VE*	0.64	0.53	0.51	0.62	0.66	0.63	0.24	0.17	0.02	0.36	0.55	0.52
	KGE*	0.77	0.09	0.45	0.63	0.73	0.69	0.37	0.29	0.05	0.45	0.50	0.56

## Discussion

This work illustrates how to use a dimensionless function that penalizes the inner balance error committed in hydrological simulations. The function can be used both to modulate the goodness of fit and as a criterion for model calibration. In order to facilitate the explanation, a lumped water balance applied in a semi-distributed manner is used as example. The concept of inner balance error can be extrapolated to more complex models, including more hydrological processes or more reservoirs, and even to distributed models. The formulation of ε can be adapted to the peculiarities of any model, and the concept would be the same. The input and output variables can change, and they can be calculated in a distributed manner, but it is still crucial to tackle the inner balance error with respect to the observed output flow, so that this error will have a physical meaning comparable with those of other catchments.

The shape of the penalty function can be changed, and several options were considered, all based on the inner balance error. One was an exponential function with two parameters that provided more flexibility for low errors, so that slight fluctuations in stored water that usually occur between wet and dry months were allowed. However, this made it more difficult to understand the nature of the penalization. Another option was a linear function, but, simulations with large balance errors would be penalized to the same extent as those with small errors, so that those catchments with large water balance errors would be confused with those with poor goodness of fit. The option of subtracting from NSE a value proportional to the inner Pbias module committed was also considered, as in Lindström [27], but it would not provide the same value for all catchments that commits a large water balance error. For these reasons, we finally selected a continuous penalty function depending on a single parameter that provides the same value (zero) when a pre-established value of error is surpassed.

It should be noted that this methodology is specifically designed to be applied in hydrological models whose structure has at least one reservoir with no capacity limitation. This is very common in most monthly scale models that simulate flow with a certain time lag. In such cases, a reservoir representative of the groundwater cycle and whose capacity is not limited is used, since the influence of groundwater in each basin can differ widely. Although this function was designed with this kind of model in mind, it could also be used for models that simulate very large basins in which the reservoir that represents soil moisture has a high capacity.

Finding the model that provides the best performance for a basin, even for different basins under different scenarios, is often the main objective to pursue in hydrological studies simulating river basins. In this kind of research it is not advisable to use a single goodness-of-fit criterion, as highlighted in this study where the abcd model provides the best performance according to the NSE. But the final decision must be made based on the greatest number of possible criteria, and applying an objective method that allows ranking the models taking into account all these criteria [53]. In these methods, the criteria used must be standardized, in order to make them comparable, and the inner balance error committed by the model (ε) could be included among them.

But, the objective of this work is not finding the best hydrological model for the study case or explaining the drawbacks of the model used. Its purpose is to emphasize the risk of selecting a hydrological model considering only one criterion of goodness of fit. In fact, this work demonstrates that making a previous study to select the hydrological model does not ensure that the selected model adequately represents the hydrology of the simulated basin. Frequently, this is due to the equifinality of the model parameters [30]. This equifinality problem is more likely to arise when the model has more parameters, since the calibration process has more degrees of freedom. In other words, it is more likely to find distinct groups of parameters that provide a similar goodness of fit. The use of a penalty function, such as the one presented, avoids selecting a set of parameters that, while providing good goodness of fit, do not meet the hypothesis that the resulting global water balance of the model should be close to zero [7]. In this sense, the penalty function introduced in the calibration solved the inner balance error in most catchments, but not in all. In four of them the problem was not totally solved. This maybe relevant because the study area includes large aquifers in carbonate rocks, so important groundwater flows, which are not controlled, may exist and should not be neglected. Therefore, despite forcing the model to have a zero-water balance, the penalty function is not capable of doing so. Thus, the application of the inner balance error in the modelling served to indicate that groundwater flows in this area should be studied in greater depth, especially if they go outside the boundaries of the Tagus River Basin to other neighbouring basins.

## Conclusions

This study has addressed two key issues in hydrology: does hydrological simulation really represent the hydrology of a given basin, and might a high goodness of fit be accompanied by an erroneous representation of the hydrology of the basin? These issues have been addressed by analysing the inner balance error committed by a hydrological model during simulation, which was subsequently applied to modulate the goodness of fit. The proposed penalty function (ϕ(ε)), which depends on the inner balance error committed during simulation, modulates the goodness of fit criteria that is maximised, as NSE, VE and KGE. For this reason, ϕ(ε) is defined as an exponential function, which depends on a parameter (α), whose values fluctuate in the interval [0–1]. The greater the value of this parameter, the higher the penalty. However, it has been seen that α-parameter values between 2 and 4 would be sufficient to ensure that internal errors between 50 and 100%, respectively, are detected. This penalty function can also be included in the objective function in order to reduce the inner balance error. To do this, it would be sufficient to multiply ϕ(ε) by the objective maximization function (such as NSE, VE or KGE). In this respect, we recommend using α = 1 in ϕ(ε) when this function is used in calibration.

## Supporting information

S1 Appendix Description of the hydrological models applied in the preliminary study. (PDF)

S2 Appendix Results of the hydrological models applied in the preliminary study. (PDF)
