# Supplementary material for: Modulation of the goodness of fit in hydrological modelling based on inner balance errors
Source: PLoS One. 2021 Nov 18;16(11):e0260117. doi: 10.1371/journal.pone.0260117 (PMC8601472; doi:10.1371/journal.pone.0260117)
Supplement: S1 Appendix — (PDF) [file pone.0260117.s001.pdf]

S1 Appendix Description of the hydrological models applied in the preliminary study

Water Balance Models Applied

The three models applied in the preliminary study simulate the hydrology by two reservoirs. One simulates the soil fast-term (direct runoff) flow by a soil moisture balance, and another recreates the long-term flow by a reservoir that represents the groundwater component in the total flow (discharges by the aquifers). The capacity in this latter reservoir has no limit. The simulations have been developed using the month as temporal interval. It should be note that all the simulation of these models needs establishing initial values for their two reservoirs: the initial soil moisture (S_0) and initial groundwater volume (G_0). These initial values are estimated in the called “take-off” of the models.

abcd model

The first step of this model is to calculate two auxiliary variables: W_t and Y_t. The first variable (W_t) represents the amount of available water in the month t, as the sum of the precipitation (P_t) and the soil moisture (S_{t-1}) at the beginning of the iteration (Eq (A.1)). And then Y_t, which depends on W_t, and the parameter a. This latter auxiliary variable represents the amount of water that can transform in evapotranspiration and/or in soil moisture (Eq (A.2)). The difference between them ($W_t - Y_t$) is called moisture excess (ME_t) (Eq (A.3)), and represents the available water that can be transform into direct runoff (Qs_t) or percolation into the groundwater reservoir *(ΔG_t)*.

$$W_t = P_t + S_{t-1} \qquad (A.1)$$

$$Y_t = \frac{W_t + b}{2a} - \sqrt{\left(\frac{W_t + b}{2a}\right)^2 - \frac{W_t b}{a}} \qquad (A.2)$$

$$ME_t = W_t - Y_t \qquad (A.3)$$

The variation of the soil moisture depends on the potential evapotranspiration (ETP_t) in the month t and on the parameter b, which represents the maximum capacity of the soil to retain water (Fig A.1). Applying this relationship, a linear differential equation is defined (Eq (A.4)). Its solution is used to determinate the soil moisture at the end of the iteration, taking Y_t as initial condition (Eq (A.5)). Once the soil moisture is calculated, the actual evapotranspiration (E_t) is determinate by a balance between these two variables (Eq (A.6)).

$$\frac{dS}{dt} = -\frac{ETP}{b} S \qquad (A.4)$$

$$S_t = Y_t exp\left(\frac{-ETP_i}{b}\right) \qquad (A.5)$$

$$E_t = Y_t + S_t \qquad (A.6)$$

The direct runoff (Qs_t) is estimated by a linear equation (Eq (A.7)) that depends on the parameter c and on the moisture excess (ME_t). The percolation (ΔG_t) into the reservoir that represents the aquifer is calculated as a water balance between moisture excess and direct runoff ($\Delta G_t = ME_t - Qs_t$) which is defined by the Eq (A.8).

$$Qs_t = (1 - c)ME_t \quad (A.7)$$

$$\Delta G_t = cME_t \quad (A.8)$$

The discharges from the aquifer (Qg_t) reservoir are a linear function that depends on the stored water at the end of the iteration (G_t), and the parameter d (Eq (A.9)). But, previously this volume of stored water is calculated by a water balance (Eq (A.10)). So, the volume at the end of the iteration is assessed by the Eq (A.11).

$$Qg_t = d \cdot G_t \quad (A.9)$$

$$\Delta G_t + G_{t-1} = G_t + d \cdot G_t \quad (A.10)$$

$$G_t = \frac{\Delta G_t + G_{t-1}}{1 + d} \quad (A.11)$$

Finally, simulated flow (Q_t) at the end of the iteration is compound by the direct runoff (Qs_t) and the discharges from the aquifer (Eq (A.12)).

$$Q_t = Qs_t + Qg_t \quad (A.12)$$

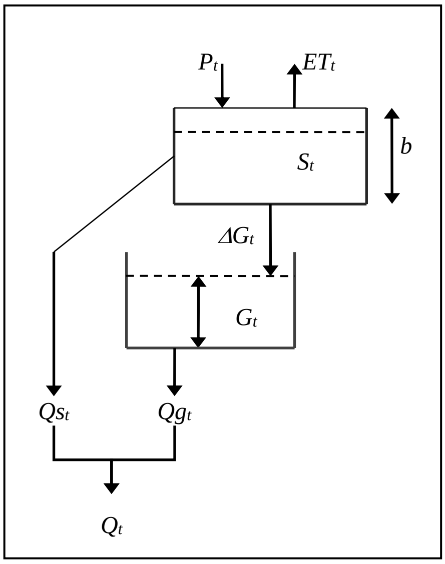

Fig A.1. Water balance diagram of the abcd model.

Thornthwaite-Matter model

The precipitation (P_t) is decomposed into direct runoff (Qs_t) and net precipitation (Pn_t) which increases the soil moisture (Eq (A.13) and Eq (A.14)). This decomposition is made linearly by a parameter α.

$$Qs_t = \alpha P_t \qquad (A.13)$$

$$Pn_t = (1-\alpha)P_t \qquad (A.14)$$

The available water in the soil, which is the net precipitation (Pn_t) plus the initial soil moisture (S_{t-1}), is compared with the potential evapotranspiration (ETP_t). If the available water is greater than potential evapotranspiration, actual evapotranspiration will be then equal to potential evapotranspiration (Eq (A.15)), and the final soil moisture at the end of the iteration is obtained by the Eq (A.16), where Φ is the maximum capacity of the soil to retain water (Fig A.2).

$$ET_t = ETP_t \qquad (A.15)$$

$$S_t = min\{S_{t-1} + Pn_t - ETP_t; \Phi\} \qquad (A.16)$$

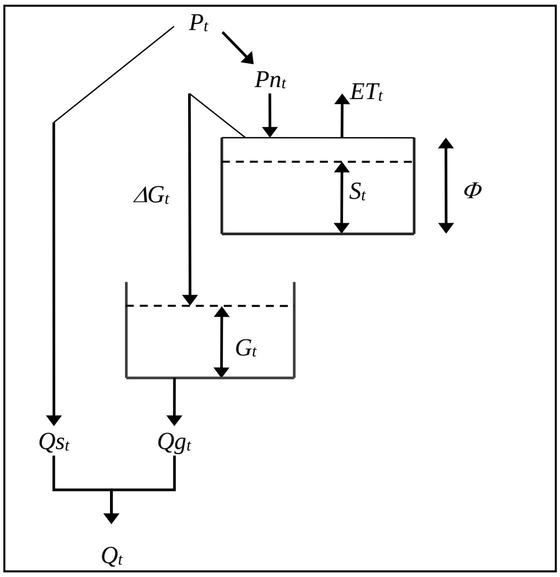

Fig A.2. Water balance diagram of the Thornthwaite-Matter model.

Otherwise, it is assumed that the soil moisture decay is the solution of a linear differential equation (Eq (A.17)). In this differential equation, the variation of the soil moisture is proportional to the initial soil moisture by the relation between ETP, Pn, and Φ.

$$\frac{dS}{dt} = -\frac{(ETP - Pn)}{\Phi} S \qquad (A.17)$$

The solution of this linear differential equation is Eq (A.18), considering as initial condition the soil moisture at the beginning of the iteration.

$$S_t = S_{t-1} exp\left(-\frac{(ETP_t - Pn_t)}{\Phi}\right) \qquad (A.18)$$

And the actual evapotranspiration is calculated now by the water balance of the Eq (A.19).

$$ET_t = S_{t-1} - S_t + Pn_t \qquad (A.19)$$

Once actual evapotranspiration is calculated, then a soil moisture balance is made in order to identify if there is deficit moisture (DM_t) (Eq (A.20)). When deficit moisture is zero, it is considered that soil is saturated and there is percolation (ΔG_t). This percolation is calculated by a water balance between the inputs and outputs in the soil, considering that, at the end of the iteration, the soil moisture is maxim and coincides with Φ (Eq (A.21)). Otherwise, if deficit moisture is greater than zero, then there is no percolation to the aquifer reservoir.

$$DM_t = \Phi - S_t \qquad (A.20)$$

$$\begin{cases} DM_t = 0 & \rightarrow \quad \Delta G_t = Pn_t - ETP_t + S_{t-1} - \Phi \\ DM_t > 0 & \rightarrow \quad \Delta G_t = 0 \end{cases} \qquad (A.21)$$

The discharges from the aquifer reservoir are simulated by a linear equation that depends on the parameter λ and the total available groundwater in the iteration ($G_{t-1} + \Delta G_t$) (Eq (A.22)). So, in order to accomplish the water balance, the groundwater in the aquifer reservoir at the end of the iteration (G_t) is the total available groundwater in the iteration multiplied by $(1 - \lambda)$ (Eq (A.23)).

$$G_t = \lambda(G_{t-1} + \Delta G_t) \qquad (A.22)$$

$$Qg_t = (1 - \lambda)(G_{t-1} + \Delta G_t) \qquad (A.23)$$

Finally, as abcd model, the simulated flow (Q_t) at the end of the iteration are compound by the sum of direct runoff (Qs_t) and discharges by the aquifer (Qg_t) (Eq (A.24)).

$$Q_t = Qs_t + Qg_t \qquad (A.24)$$

GR2 model

The first step in this model is to transform two variables (precipitation (P_t) and potential evapotranspiration (ETP_t)) in order to create a new variable (U_t) with the same dimensions. This transformation depends on the parameter x_1 (Eq (A.25))

$$U_t = \frac{(x_1 P_t)(x_1 ETP_t)}{\left(\sqrt{x_1 P_t} + \sqrt{x_1 ETP_t}\right)^2} \qquad (A.25)$$

This new variable (U_t) is subtracted to these transformed variables ($x_1 P_t$, $x_1 ETP_t$), creating a new variables related with the precipitation and potential evapotranspiration that will condition the soil moisture (S). These new variables end up being the net precipitation and the actual evapotranspiration (Pn_t, En_t) at the iteration (Eqs (A.26) and (A.27)).

$$Pn_t = x_1 P_t - U_t \qquad (A.26)$$

$$En_t = x_1 ETP_t - U_t \qquad (A.27)$$

At the beginning of the iteration, the soil moisture will increase ($S_{t-1} \rightarrow S_{1t}$) by the Eq (A.28). This depends on the parameter A, which represents the maximum capacity of the soil to retain water (Eq (A.29)) and a new transformed variable V_t. The latter depends on the net precipitation and the same parameter A (Fig A.3).

$$S_{1t} = \frac{S_{t-1} + A \cdot V_t}{1 + \frac{S_{t-1} \cdot V_t}{A}} \qquad (A.28)$$

$$V_t = tanh\left(\frac{Pn_t}{A}\right) \qquad (A.29)$$

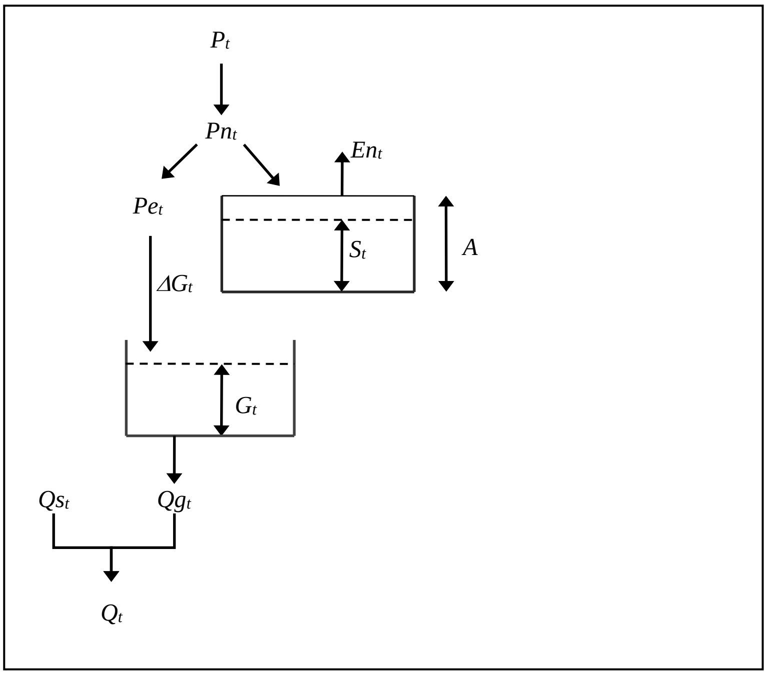

Fig A.3. Water balance diagram of the GR2 model.

Next, the soil moisture change again ($S_{1t} \rightarrow S_{2t} \rightarrow S_t$) by the effect of the actual evapotranspiration and parameter A (Eq (A.30) and Eq (A.31)).

$$S_{2t} = \frac{S_{1t}(1 - W_t)}{1 + W_t\left(1 - \frac{S_{1t}}{A}\right)} \qquad (A.30)$$

$$W_t = tanh\left(\frac{En_t}{A}\right) \qquad (A.31)$$

The amount of net precipitation (Pn_t) that has not accumulated as soil moisture (Pe_t) is determined by a water balance (Eq (A32)). This variable will contribute to the direct runoff by a parameter α, and the remaining serves to the increase the stored water in the aquifer reservoir (Eqs (A.33) and (A.34)).

$$Pe_t = Pn_t - (S_{2t} - S_{t-1}) \qquad (A.32)$$

$$Qs_t = \alpha Pe_t \quad (A.33)$$

$$\Delta G_t = (1 - \alpha) Pe_t \quad (A.34)$$

Then, the stored water in the aquifer will increase (Eq (A.35)), and the discharges of the aquifer are modelled by a lineal reservoir by the x_2 parameter (Eq (A.36)).

$$G_t = G_{t-1} + \Delta G_t \quad (A.35)$$

$$Qg_t = x_2 G_t \quad (A.36)$$

Finally, as previous models, simulated flow (Q_t) at the end of the iteration are compound by the direct runoff (Qs_t) and the discharges by the aquifer (Qg_t) (Eq (A.37)).

$$Q_t = Qs_t + Qg_t \quad (A.37)$$
