# Supplementary material for: Modulation of the goodness of fit in hydrological modelling based on inner balance errors
Source: PLoS One. 2021 Nov 18;16(11):e0260117. doi: 10.1371/journal.pone.0260117 (PMC8601472; doi:10.1371/journal.pone.0260117)
Supplement: S2 Appendix — (PDF) [file pone.0260117.s002.pdf]

S2 Appendix. Results of the hydrological models applied in the preliminary study

In this appendix a summary of the results of applying three hydrological models in the study area (The Headwater of the Tagus River Basin) is presented. Both the data used and the calibration process followed are those described in the section 2 on the paper. The NSE is used as an objective function with the transformed flow (by means of the square root). The presented NSE value has been calculated by undoing the square root transformation.

The obtained NSE values are presented in the following Tables (Tables B.1 - B.3). Among the three hydrological models used, the abcd model was the one that provided the highest and most consistent NSE values, both in calibration and in validation periods (Table B.1). While both the Thornthwaite-Mather model (Table B.2) and the GR2 model (Table B.3) provided lower values, above all in the validation period.

Table B.1. NSE results of applying the abcd model in the study area.

**Catchment code**	**3001**	**3030**	**3268**	**3005**	**3006**	**3045**	**3172**	**3173**	**3186**	**3201**	**3041**	**3043**
**Calibration**	0.77	0.39	0.69	0.71	0.75	0.82	0.73	0.76	0.57	0.84	0.74	0.86
**Validation**	0.59	0.34	0.49	0.59	0.68	0.76	0.63	0.49	0.70	0.77	0.75	0.70

Table B.2. NSE results of applying the Thornthwaite-Mather model in the study area.

**Catchment code**	3001	3030	3268	3005	3006	3045	3172	3173	3186	3201	3041	3043
**Calibration**	0.75	0.13	0.54	0.71	0.74	0.69	0.25	0.44	0.12	0.66	0.70	0.85
**Validation**	0.62	-0.55	-0.01	0.55	0.64	0.70	0.14	0.12	-1.00	0.59	0.70	0.56

Table B.3. NSE results of applying the GR2 model in the study area.

**Catchment code**	3001	3030	3268	3005	3006	3045	3172	3173	3186	3201	3041	3043
**Calibration**	0.72	0.35	0.52	0.45	0.75	0.76	0.48	0.51	0.51	0.66	0.71	0.87
**Validation**	0.65	0.34	0.30	0.09	0.65	0.67	0.50	0.40	0.61	0.63	0.73	0.85
